# Revisiting the phylogeography, demography and taxonomy of the frog genus *Ptychadena* in the Ethiopian highlands with the use of genome-wide SNP data

**DOI:** 10.1371/journal.pone.0190440

**Published:** 2018-02-01

**Authors:** Jacobo Reyes-Velasco, Joseph D. Manthey, Yann Bourgeois, Xenia Freilich, Stéphane Boissinot

**Affiliations:** 1 New York University Abu Dhabi, Saadiyat Island, Abu Dhabi, United Arab Emirates; 2 Department of Biology, Queens College, City University of New York, Flushing, New York, United States of America; National Cheng Kung University, TAIWAN

## Abstract

Understanding the diversification of biological lineages is central to evolutionary studies. To properly study the process of speciation, it is necessary to link micro-evolutionary studies with macro-evolutionary mechanisms. Micro-evolutionary studies require proper sampling across a taxon’s range to adequately infer genetic diversity. Here we use the grass frogs of the genus *Ptychadena* from the Ethiopian highlands as a model to study the process of lineage diversification in this unique biodiversity hotspot. We used thousands of genome-wide SNPs obtained from double digest restriction site associated DNA sequencing (ddRAD-seq) in populations of the *Ptychadena neumanni* species complex from the Ethiopian highlands in order to infer their phylogenetic relationships and genetic structure, as well as to study their demographic history. Our genome-wide phylogenetic study supports the existence of approximately 13 lineages clustered into 3 species groups. Our phylogenetic and phylogeographic reconstructions suggest that those endemic lineages diversified in allopatry, and subsequently specialized to different habitats and elevations. Demographic analyses point to a continuous decrease in the population size across the majority of lineages and populations during the Pleistocene, which is consistent with a continuous period of aridification that East Africa experienced since the Pliocene. We discuss the taxonomic implications of our analyses and, in particular, we warn against the recent practice to solely use Bayesian species delimitation methods when proposing taxonomic changes.

## Introduction

The impact of geographical and ecological variation on species diversification is central to evolutionary studies and conservation biology [1–3]. Approaches that link rates of diversification among lineages with several biogeographic and abiotic landscape characteristics, such as climate or topography, have strongly improved our understanding of the general trends and processes that drive speciation [4, 5]. A proper assessment of speciation mechanisms requires connecting macro- and micro-evolutionary approaches [1, 4, 6]. Macro-evolutionary approaches are popular in speciation studies, and most of our current knowledge of species radiations takes root in them [7, 8]. In contrast, microevolutionary studies are less common, requiring large sample sizes and adequate geographical coverage to properly assess diversity within and between populations.

Speciation is a complex process by which an ancestral population becomes two or more distinct taxa. It is commonly described as a continuum encompassing all stages of divergence, from panmictic populations to irreversibly isolated species [9]. The stage in this continuum at which two populations are divergent enough to be called species is a much-debated topic. Multiple species concepts are currently used for different taxonomic groups, and are not necessarily applied to similar stages in the speciation process [9, 10]. Because speciation is a continuum, we need to assess intraspecific genetic variation at multiple points in the speciation process to fully understand it. [9, 11]. The increasing availability of next-generation sequencing facilitates combining techniques that bridge the gap between macro- and micro-evolutionary scales [9, 12]. This expansion is critical for both basic biological research [1, 9, 13] and conservation [10]. For example, using genomic tools to study intra-specific diversity may reveal cryptic diversity in lineages for which morphology alone cannot predict the existence of relevant conservation units [14].

Here we use a group of frogs from the Ethiopian highlands (genus *Ptychadena*) as a model to study the speciation process at several stages, from intraspecific genetic variation to interspecific genetic divergence. The genus *Ptychadena* is widespread across Africa, and is one of a few groups of frogs that managed to colonize oceanic islands [15, 16] as well as some of the highest mountains in Africa [17]; these characteristics make the genus *Ptychadena* unique among African amphibians. In the Ethiopian Highlands, these frogs have colonized a variety of habitats, ranging from perturbed cultivation fields to forests and moorlands at more than 3,500 m [17].

The taxonomy of *Ptychadena* in Ethiopia is problematic [18]. In particular, they have a relatively conserved morphology as well as local color polymorphism, making it difficult to define diagnostic characters between the multiple species in the genus. A recent phylogenetic study of Ethiopian *Ptychadena* [19] found surprisingly high levels of genetic differentiation between populations of the endemic *P*. *neumanni*. Freilich *et al*. [19] used multiple nuclear and mitochondrial DNA (mtDNA) loci to infer phylogenetic relationships between populations and to estimate the number of species of this genus in Ethiopia. They found that *P*. *neumanni* is a complex of eight different species that includes five undescribed taxa as well as the species *P*. *erlangeri*, *P*. *nana*, and *P*. *cooperi*. They also found that these species have defined elevational ranges and are restricted to specific habitats. However, the study by Freilich *et al*. did not allow for a thorough investigation of intraspecific variation and demographic history, because of the limited phylogenetic information available in most nuclear loci they used.

In this study, we combine phylogenetic and population genomic approaches to study the phylogeography, diversity, and demography of the *P*. *neumanni* species complex in the Ethiopian Highlands. Additionally, we aimed to identify any environmental and geographic effects shaping the evolutionary history and speciation process in this group of frogs. The *P*. *neumanni* complex represents a useful model system to study the speciation process in one of the most unique areas of Africa. We accomplished our research goals with the use of extensive sampling across the Ethiopian Highlands and by sequencing thousands of putatively unlinked nuclear loci obtained with double digest restriction site associated DNA sequencing (ddRAD-seq). We also address important taxonomic issues in this and other groups of vertebrates that have become more prevalent in recent years, especially with the increased use and misuse of coalescent species delimitation methods.

## Materials and methods

### Sample collection/Ethics statement

Our study was approved by the relevant Institutional Animal Care and Use Committee (IACUC), at the New York University School of Medicine. Frogs were sampled according to permits DA31/305/05, DA5/442/13 and DA31/454/07, provided by the Ethiopian Wildlife Conservation Authority. We conducted multiple field collecting trips across Ethiopia between 2010 and 2016. Our efforts focused on the Ethiopian highlands, with sampling on both sides of the Great Rift Valley (GRV). However, we also conducted trips to multiple lowland areas. We collected most individuals by hand at night, usually when frogs were calling from bodies of water such as rivers, creeks, ponds, cattle tanks, etc. We also collected many juveniles and tadpoles in multiple developmental stages with the use of nets. We photographed each individual, and performed euthanasia with ventral application of benzocaine. We euthanized tadpoles by submersion in 10% ethanol. We sampled muscle or liver tissue for each specimen and preserved it in either 95% ethanol, cell lysis buffer, or RNAlater (Invitrogen). We fixed adult and juvenile specimens with injection of 10% formalin and later preservation in 70% ethanol, while we preserved tadpoles in 10% formalin. We deposited all specimens at the Zoology Museum of the University of Addis Ababa, Ethiopia. Tissue samples are deposited at the Vertebrate Tissue Collection, New York University Abu Dhabi (NYUAD). In total, we collected 289 individuals from 129 localities (S1 Table and S1–S3 Figs).

In this study we follow the nomenclature of Freilich *et al*. [19]. Since these authors did not perform a detailed morphological analysis of their samples, they did not formally describe the putative species they discovered, but instead assigned numbers to each lineage (eg. *P*. cf. *neumanni 1*, *P*. cf. *neumanni 2*, etc.). Recently Smith *et al*. [20] assigned new names to multiple *Ptychadena* from Ethiopia. However, the new names lack appropriate diagnostic characters as defined by the International Commission on Zoological Nomenclature [21]. As a result, all the proposed new species’ names should be considered as *nomina nuda*, according to the International Commission on Zoological Nomenclature [21] and are disregarded by us in the present paper. See Discussion for a more detailed description regarding issues with the taxonomy of this group.

### DNA extraction and PCR amplification

We used one of several methods to extract genomic DNA from tissue samples: DNeasy blood and tissue kit (Qiagen, Valencia, CA), using Serapure beads [22], or by standard potassium acetate extraction. We measured DNA concentration with a Qubit fluorometer (Life Technologies) so that we could standardize all DNA sample concentrations.

Because it is difficult to assign tadpoles to a taxonomic group while in the field, we barcoded most tadpoles we collected, as well as all adult specimens and juveniles. We sequenced a fraction of the 16s rRNA mitochondrial gene with the primers LX12SN1a and LX16S1Ra [23] or the 16Sar and 16Sbr primers [24]. We amplified the 16s gene with polymerase chain reaction (PCR) using Taq polymerase (Invitrogen) in reaction volumes of 25 μl. We performed the PCR with an initial denaturation temperature of 96°C (2 minutes), a subsequent 35 cycles of denaturation at 94°C (15 seconds), annealing at 50°C (1 minute), and an extension at 72°C (2 minutes), followed by a final extension at 72°C (10 minutes). We shipped the unpurified PCR products for sequencing at BGI Tech Solutions (Hong Kong).

### Genetic barcoding and phylogenetic analysis of mtDNA

We used the 16s dataset of [19] to assign specimens to a particular *Ptychadena* mtDNA lineage. We manually trimmed each sequence in the program Geneious v9.1.6 (Biomatters Ltd., Auckland, NZ) using the raw chromatogram files. We included an additional 87 samples of Ethiopian *Ptychadena* obtained from Genbank (S2 Table). We performed nucleotide alignments in MAFFT version 7 [25] and created a final 16s alignment of 516 bp. We then used the Bayesian information criterion (BIC), implemented in PartitionFinder v1.1.1 [26], to select the best-fit model of nucleotide evolution for our dataset.

We performed Bayesian phylogenetic inference (BI) using Mr. Bayes v3.2.2 [27] on the CIPRES Science gateway server [28]. The BI analysis consisted of four runs of 10^7^ generations, with four chains (three heated, one cold) and sampled every 1,000^th^ generation. We checked for convergence of each run in Tracer v 1.6 [29] by visually assessing overlap in likelihood and parameter estimates between runs, as well as effective sample sizes and potential scale reduction factor (PSRF) value estimates for each run. Based on the PSRF, individual runs converged by 10^5^ generations. We therefore discarded the first 25% of each run as burn-in, combined the runs and then visualized the final tree in FigTree v1.4.2 (http://tree.bio.ed.ac.uk/software/figtree/).

### ddRADseq library preparation and sequencing

We used ddRAD-seq to obtain genome-wide SNPs of many individuals of Ethiopian *Ptychadena*. We digested genomic DNA for 7 hours at 37°C with the enzymes SbfI and MspI [30]. We then purified DNA fragments using Serapure beads [22], ligated adapters with attached barcodes [31] and pooled samples in groups of eight (number of unique barcodes; S3 Table). We size-selected each pooled library of barcoded samples between 400 and 550 bp using a Pippin Prep (Sage Science, Beverly, MA, USA), and then amplified the size-selected pooled libraries using PCR to attach unique Illumina indices [31] (S3 Table). We determined fragment size and concentration for each library on a Bioanalyzer 7500 with a high sensitivity DNA chip (Agilent, Santa Clara, CA, USA), and checked library quantity with quantitative PCR. We then pooled all libraries and sequenced them with an Illumina HiSeq2500 (100 bp paired-end reads) at the Genome Core Facility of New York University Abu Dhabi, United Arab Emirates.

### ddRADseq data analyses

We trimmed restriction sites with the FASTX Toolkit [32] and then used ipyrad 0.6.17 [33] to assemble loci *de novo* and create SNP datasets. In ipyrad, we discarded all sequences with an average *phred* score offset of less than 33, and with more than 5 low quality bases per read. We then used an 85% clustering threshold and kept all other parameters at default values. We required each locus to be present in at least 50% of all individuals. Because preliminary results showed great divergence between the different species of *Ptychadena*, as well as few shared loci between species, we performed a second run of the ipyrad pipeline with the same parameters as above to create three different datasets, each including only samples that grouped with the *cooperi*, *erlangeri*, or *nana* groups as defined by [19].

After quality filtering, we retained a total of ~158 million sequencing reads, with highly variable coverage across individuals (mean = 1.60 million, sd = 0.6 million, S3 Table). This resulted in a mean of ~11,400 RAD-tags (sd = 2,000) per individual. Of the three SNP datasets, we obtained between 800 and 2918 polymorphic loci and between 28,000–36,000 SNPs (S3 Table).

### Phylogenetic analysis of genome-wide SNP data

We used the Bayesian Information Criterion (BIC) in PAUP* v.4.0.a151 [34] to estimate the best model of evolution for our concatenated ddRADseq dataset (GTR + I + G). We then inferred evolutionary relationships using maximum likelihood (ML) implemented in RAxML v8 [35]. We performed RAxML with rapid bootstrapping, implemented in the CIPRES portal [28]. We ran RAxML with all samples of *Ptychadena* (n = 97) as well as for each of the *cooperi*, *erlangeri*, and *nana* species groups.

### Species-tree estimation of SNP data

We used SVDquartets [36], implemented in PAUP* v.4.0.a151 [34], to infer phylogenetic relationships between the different genetic clusters identified in the STRUCTURE and ML analyses. We ran the SVDquartets analysis separately on each one of the different species groups (*cooperi*, *nana*, and *erlangeri*). In SVDquartets, we used all possible quartets for species tree inference, with 100 bootstrap replicates to assess support. Additionally, we visualized conflicting phylogenetic signal from the SNP data by constructing phylogenetic networks from the SNP data with the use of the NeighborNet algorithm in SplitsTree 4 [37, 38] with heterozygous sites averaged.

### Population structure and nucleotide diversity

In order to examine genetic structure among individuals and species without *a priori* inferences, we used the STRUCTURE software [39]. In this case we used a single random SNP from each RADseq locus. We performed this analysis separately on each of the three different species groups of *P*. *neumanni* (*cooperi*, *nana*, and *erlangeri*) as only a small number of loci were recovered across all samples (S3 Table). For each group, we initially performed a single STRUCTURE run to infer lambda, with the number of populations (k) set to one. We then performed STRUCTURE using the admixture model and correlated allele frequencies, a constant value of lambda, for a burn in period of 50,000 generations, followed by 50,000 additional generations. We ran STRUCTURE using a range of k values (1–12) with five replicates each. With the STRUCTURE output, we used the ΔK method [40] to identify the most likely number of clusters on each of the different species groups. We also followed the recommendation of Meirmans [41] to use the highest number of genetic clusters that makes biological sense. Using the SNP dataset, we computed the amount of fixed, shared, and private polymorphisms for each population or species. We computed this separately for each of the species groups recovered in the ML analysis.

### Demography

To assess whether genetic clusters displayed evidence of variation in past population sizes, we estimated parameters for a two-epoch demographic model. We used the likelihood framework implemented in fastsimcoal2.5 [42, 43] which is based on the allele frequency spectrum (AFS). The model included five parameters (three effective population sizes allowed to change at two different times in the past). We included all population clusters with at least four individuals in the analysis.

We ran the ipyrad pipeline including only individuals from species or populations for which we had at least four individuals, and only included loci found in at least 50 percent of those individuals. We projected the folded AFS down in each cluster to increase the number of segregating sites with the use of a custom python script (available at https://github.com/isaacovercast/easySFS). We obtained parameters with the highest likelihood after 40 cycles of the algorithm, starting with 50,000 coalescent simulations per cycle, and ending with 100,000 simulations. We replicated this procedure 50 times and retained the set of parameters with the highest final likelihood as the best point estimate. We estimated 95% confidence intervals (CI) using a non-parametric bootstrap procedure, creating 100 pseudo-observed AFS by sampling with replacement from the observed allele frequency spectrum and estimating parameters as described before.

As a secondary estimate of demographic scenarios, we also used Stairway v2., a software that fits a flexible multi-epoch demographic model similar to a skyline plot and relies on the AFS [44]. This method facilitates exploratory analyses by not making assumptions about the number of past bottlenecks or expansions. We used the default parameters as recommended, running 200 bootstrap replicates to estimate 95% confidence intervals.

## Results

### Estimates of evolutionary relationships

The mitochondrial locus 16s proved useful to distinguish individuals between the different putative species and species groups of *Ptychadena*. However, many branches received low posterior support and proved of poor use for inferring phylogenetic relationships, but very useful as a barcoding tool (S4–S6 Figs).

**Fig 1:**
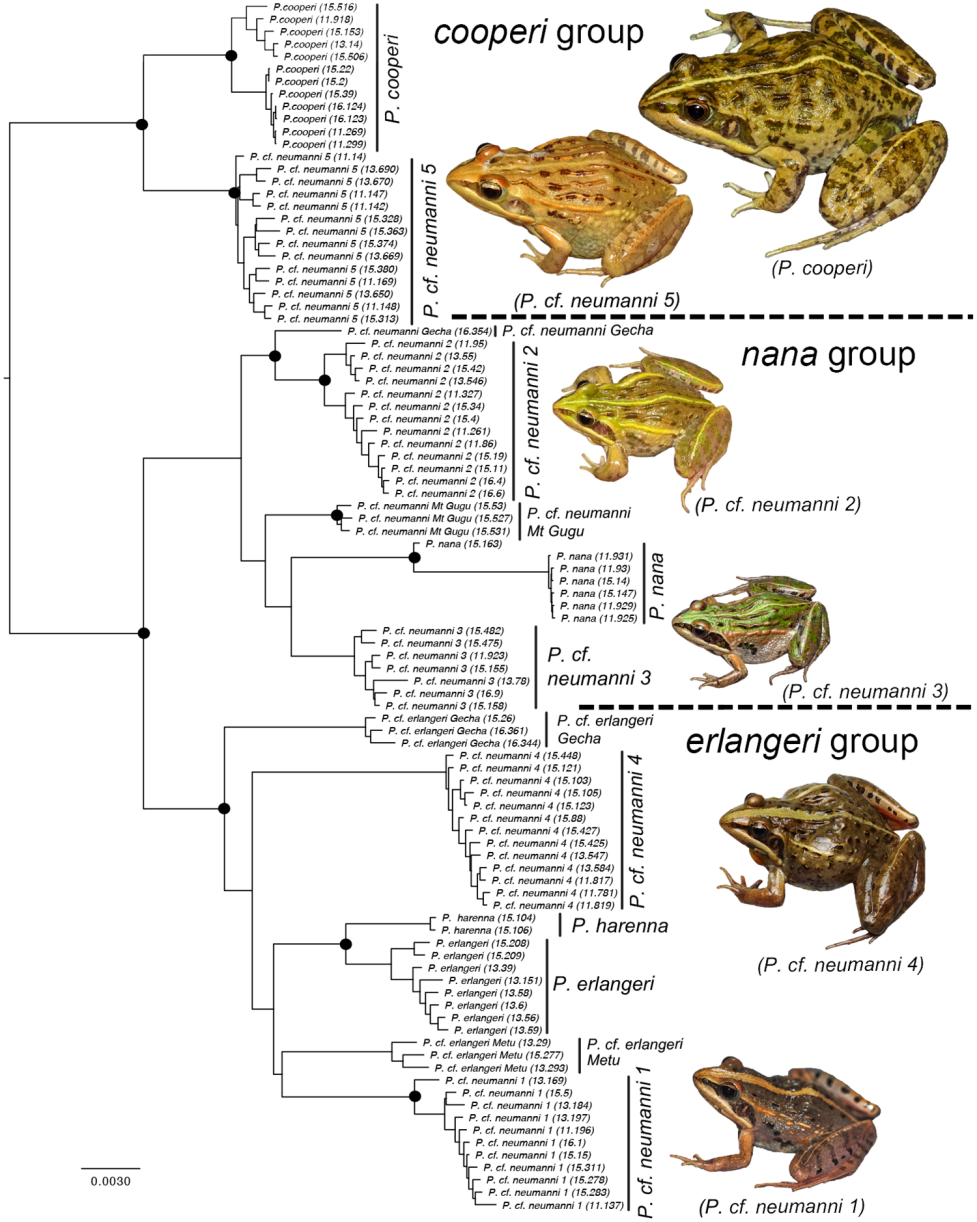
Maximum likelihood estimate (ML) of phylogenetic relationships in the *Ptychadena neumanni* species complex, inferred from the ddRADseq concatenated SNP dataset. Black circles represent nodes with >95% bootstrap support. Inset frogs are representatives of each species group. Frogs are relatively at the same scale.

The ML analysis of the concatenated SNP dataset recovered three well supported groups (Fig 1), which partially correspond to those recovered by Freilich et al. [19]. The majority of the deeper nodes in our phylogeny had strong support, with most nodes showing 100% bootstrap support. We recovered *Ptychadena cooperi* and *P*. cf. *neumanni 5* as sister to one another (*cooperi* group; Fig 1), and together they form the sister group to all other *Ptychadena* analyzed. These two species are separated by the deep gorges of the Blue Nile River in northern Ethiopia. Individuals of *P*. *cooperi* from the east and west of the Great Rift Valley form reciprocally monophyletic groups, suggestive of genetic structure. Hereafter, we refer to this lineage as the *cooperi* group.

We recovered all remaining members of the *P*. *neumanni* species complex as a monophyletic group composed of two main lineages. The first one, which we refer to from now on as the *nana* group, consists of species that generally inhabit the plateaus and mountain habitats above 2,500m. It is composed of *P*. *nana*, *P*. cf. *neumanni 2* and *P*. cf. *neumanni 3*, as well as two novel genetic lineages (Fig 1). The most basal split in this lineage separates three mountain populations from the eastern side of the GRV from *P*. cf. *neumanni 2*, which is the only taxon in this group to be found on both sides of the GRV, and a single individual from the vicinity of the town of Gecha, locate in SW Ethiopia. The individual from Gecha (*P*. cf. *neumanni* Gecha) was collected in the southwestern part of the country, in a habitat (tropical forest) and at an elevation (2,200m) unusual for this group, which is usually found in grasslands above 2,500m. The three taxa endemic to the eastern highlands are *P*. cf. *neumanni 3* (from the western part of the Bale mountains), *P*. *nana* (from the eastern part of the Bale mountain and the plateau east of Bale) and a novel taxon which seem restricted to Mount Gugu in the northeastern most part of the Arsi plateau (*P*. cf. *neumanni* Mt Gugu). Although this group is well supported, relationships among the individual lineages are not, particularly for the populations of the eastern highlands (Fig 1).

The last group (*erlangeri* group) consists of six distinct lineages, which are found in tropical forests or grasslands at elevations usually below 2,500m. It is composed of *P*. *erlangeri*, *P*. *harenna*, *P*. cf. *neumanni 1* and *P*. cf. *neumanni 4*, as well as two newly discovered populations (Fig 1). Three lineages are restricted to the west of the GRV, two to the east and one species (*P*. *erlangeri sensu stricto*) is found on both sides. The western lineages include the widespread grassland species *P*. cf. *neumanni 1* and two novel lineages from the forests of the southwest, one found in the forests from the town of Gecha to near Jimma (*P*. cf. *erlangeri* Gecha), and a lineage distributed between the towns of Metu and Bedele (*P*. cf. *erlangeri* Metu). In the east, *P*. cf. *neumanni 4* is widely distributed in the forests covering the southern flanks of the eastern highlands. We recovered *P*. *erlangeri* from both sides of the GRV as sister to two individuals tentatively assigned to *P*. *harenna* from the Harenna forest in SE Ethiopia. We tentatively assigned these two individuals to *P*. *harenna* as the 16s phylogeny group them with topotypic samples of this species from Genbank and because they were collected near the type locality.

**Fig 2:**
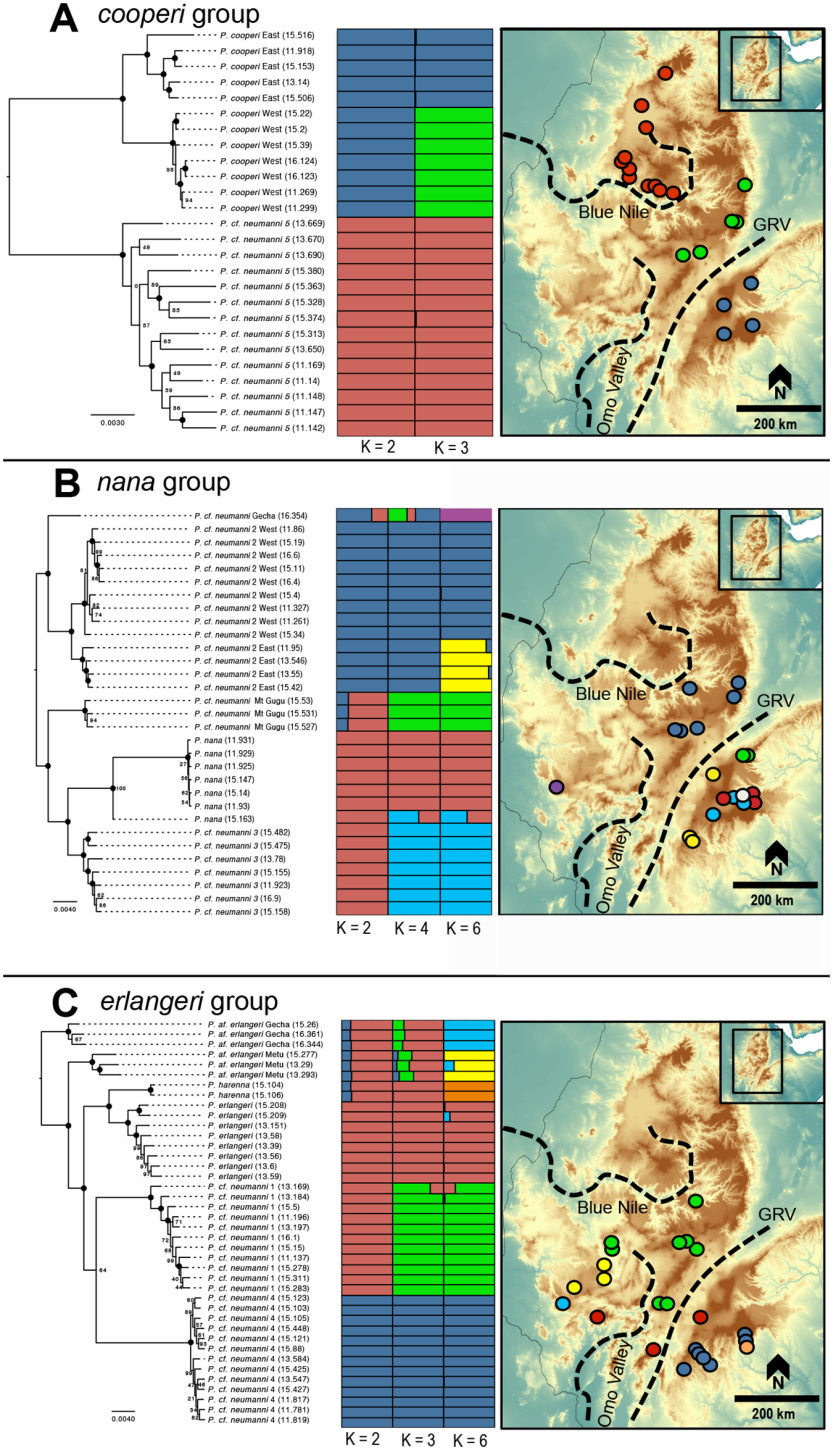
ML phylogeny, STRUCTURE plot, and sampling localities for each of the three species groups. A) *cooperi* group. B) *nana* group. C) *erlangeri* group. Left panel—ML phylogenetic estimate of concatenated ddRADseq SNP data. Central panel—STRUCTURE plot for each of the species groups obtained using a single SNP per locus. Right panel—Sampling localities for individuals used in the ddRADseq study.

Because only a few loci were shared across all samples when we included all samples of Ethiopian *Ptychadena*, we performed the ML analyses for each of the three species groups separately (Fig 2, left panel). Because we did not include any outgroups (in order to recover as many loci as possible), these trees were rooted with the most divergent member of each group, as inferred by the ML analysis of all samples. These analyses are mostly in agreement with the ML analysis of all taxa, and the support values for the nodes are usually higher. The only differences between the topologies are the placement of *P*. cf. *erlangeri* Metu, which is recovered as sister to all other members of the *erlangeri* group, with the exception of *P*.cf. *erlangeri* Gecha (vs. sister to *P*. cf. *neumanni* 1), and the placement of *P*. cf. *neumanni* 4, which was recovered as sister to *P*. cf. *neumanni* 1 (vs. sister to most other lineages), but with low support (Fig 2c).

### Population structure

We recovered multiple genetic clusters in each of the *Ptychadena* species groups using the STRUCTURE analyses (Fig 2, center panel). In the *cooperi* group, we found the most support for two genetic clusters, which correspond to *P*. cf. *neumanni 5* and *P*. *cooperi*. If three genetic clusters were assumed, populations of *P*. *cooperi* east and west of the GRV were split from each other (Fig 2a, center panel).

We found the strongest support for six genetic clusters in the *nana* group (Fig 2b, center panel); Here, we found distinct genetic clusters for *P*. cf. *neumanni 3*, *P*. *nana*, *P*. cf. *neumanni* Mt. Gugu, and three different groups of *P*. cf. *neumanni 2*. The genetic clusters of *P*. cf. *neumanni 2* correspond to the populations on each side of the GRV as well as the lone individual from the town of Gecha in southwestern Ethiopia (*P*. cf. *neumanni* Gecha). We found low levels of admixture between genetic clusters of *P*. cf. *neumanni 2* across the GRV but none between the individual from Gecha and other populations. One individual of *P*. *nana* (15.163) appears to be a hybrid between this species and *P*. cf. *neumanni 3*. Apart from this individual, we found little evidence for admixture between groups.

In the *erlangeri* group, six genetic clusters received the strongest support (Fig 2c, center panel), which correspond to the lineages recovered in the ML analyses. We found low levels of admixture between *P*. cf. *erlangeri* individuals from Gecha and Metu in southwestern Ethiopia and between *P*. *erlangeri* and *P*. cf. *neumanni 1* in a single individual (13.169; Fig 2c).

### Species-trees and SplitsTree networks

**Fig 3:**
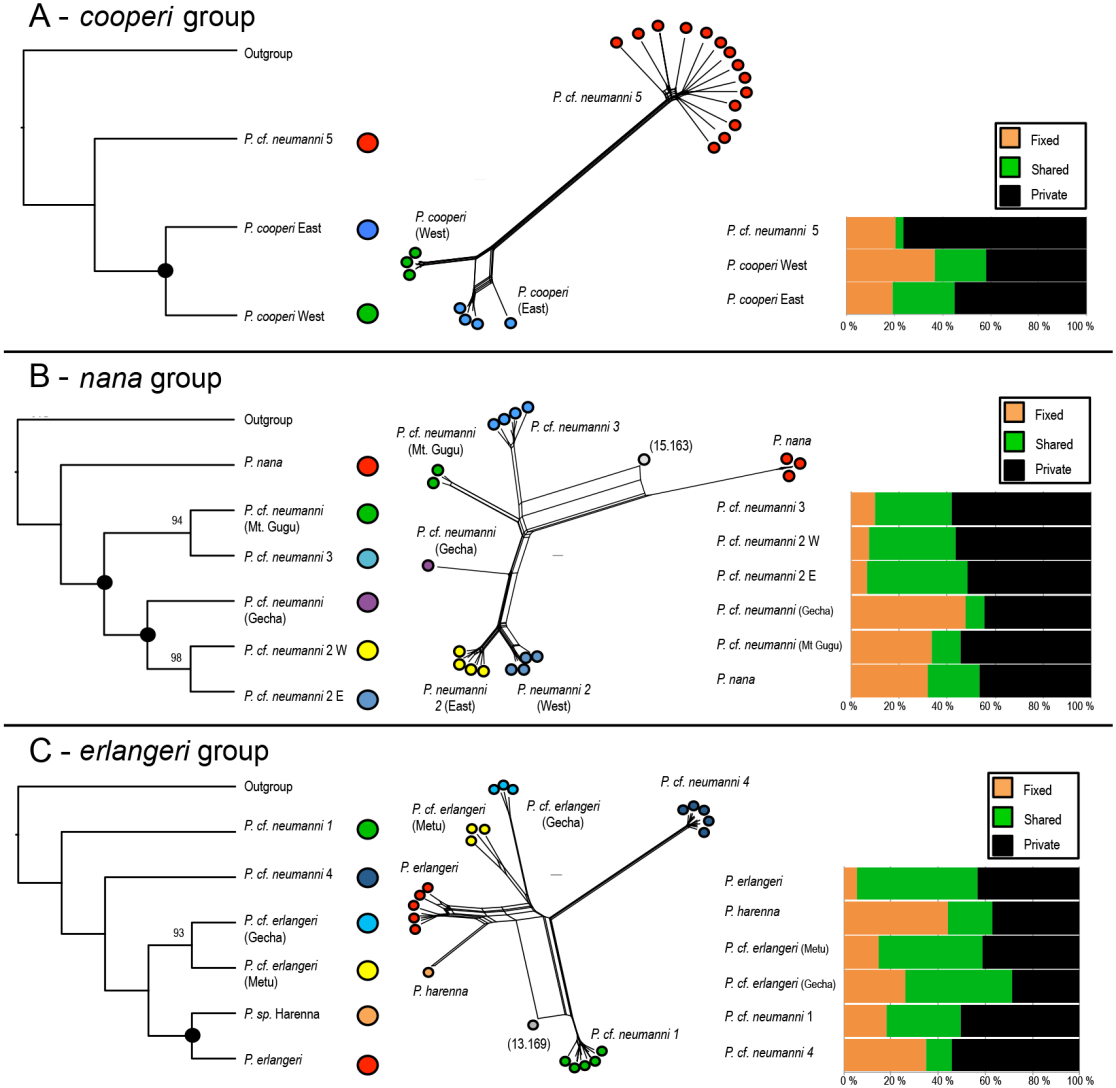
Species tree estimates, SplitsTree networks, and polymorphisms in the *P*. *neumanni* species complex. A) *cooperi* group. B) *nana* group. C) *erlangeri* group. Left panel—Species-tree estimate computed in SVDquartets for each of the species groups. Central panel—SplitsTree networks for each SNP dataset. Right panel—Distribution of fixed, shared, and private polymorphisms for each species group.

Using multispecies coalescent species-tree analyses in SVDquartets, we recovered evolutionary relationships that were slightly different from those obtained in the RAxML analysis (Fig 3, left panel). No differences were found between the topologies of the SVDquartets analysis and the ML analysis of the *cooperi* group (Fig 3a, left panel). In the *nana* group, the SVDquartets tree places *P*. *nana* as the earliest split in the group (Fig 3b, left panel), while it was sister to *P*. cf. *neumanni 3* in the ML analyses (Figs 1 and 2). In the *erlangeri* group, the SVDquartets topology differed from the ML phylogenies, as the SVDquartets recovered *P*. cf. *neumanni* 1 as the earliest split in this species group (vs. *P*. cf. *erlangeri* Gecha as the earliest split in the ML analysis), followed by *P*. cf. *neumanni* 4 as the next split (vs. sister taxa to *P*. cf. *neumanni* 1 in the ML analysis). The SplitsTree network analyses of the three species groups identified very similar patterns as those obtained from the ML and STRUCTURE analyses of the same data (Fig 3, center panel).

### Nucleotide diversity and demography

Each of the different species and populations of *Ptychadena* had highly variable levels of nucleotide diversity (Fig 3, right panel; S5 Table). We found the highest number of private polymorphisms in *P*. cf. *neumanni 5* (~75%), yet private polymorphisms were abundant in all species and populations. The numbers of fixed polymorphisms differ greatly among groups and were the highest (~35% or above) for the northern population of *P*. *cooperi*, *P*. cf. *neumanni* from Gecha and Mt. Gugu, *P*. *nana*, *P*. *harenna*, *P*. cf. *erlangeri* Gecha, and *P*. cf. *neumanni* 4. Yet, all populations contained fixed differences as well as high proportions of private alleles, suggestive of high levels of differentiation.

**Fig 4:**
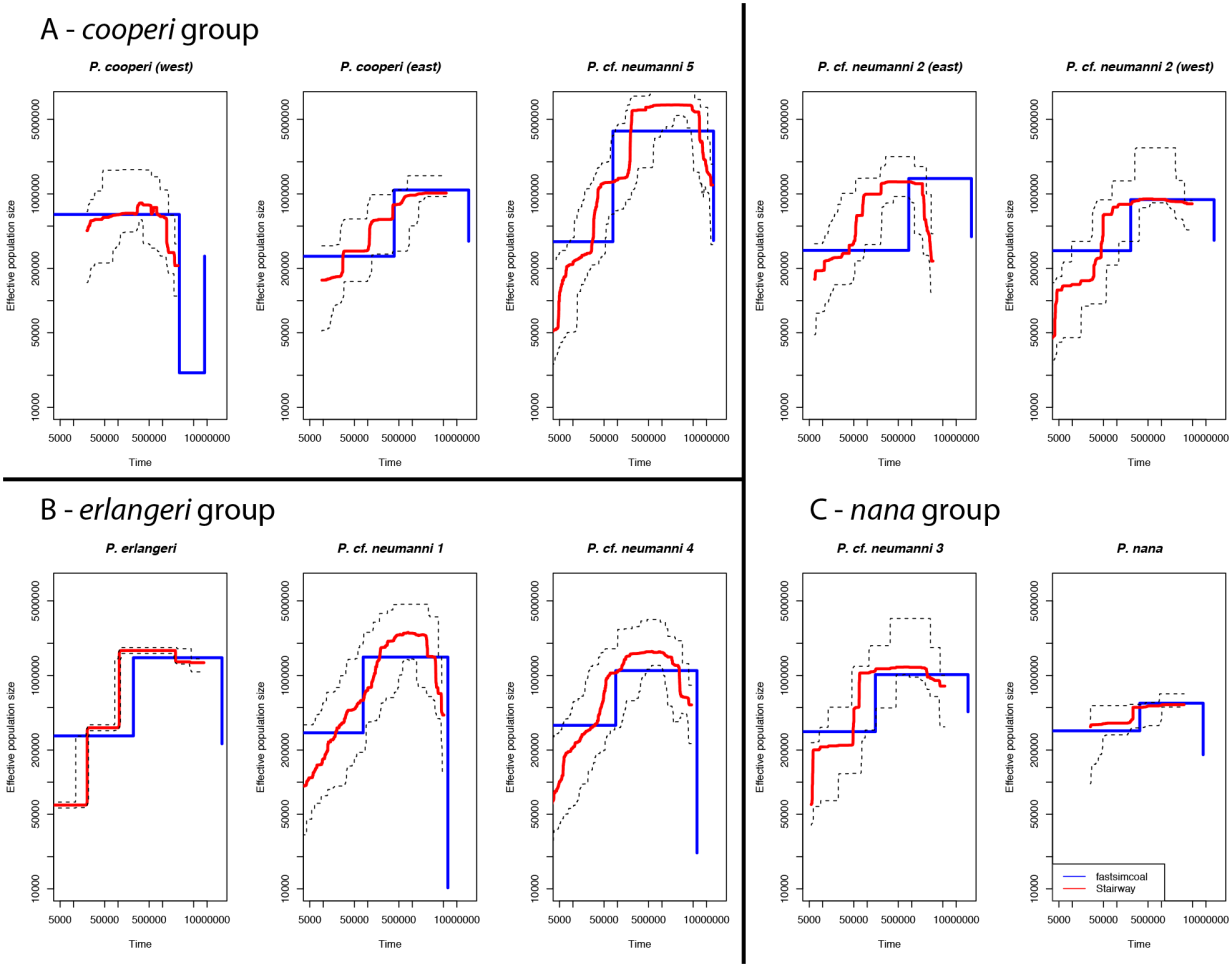
Demographic history of populations in the *P*. *neumanni* species complex. A- *cooperi* group. B–*erlangeri* group. C–*nana* group. Blue and red lines represent demographic history inferred with fastsimcoal and Stairway, respectively. Dashed lines represent confidence intervals obtained in Stairway.

We found consistent estimates of current effective population sizes and demographic trajectories between the programs fastsimcoal and Stairway (Fig 4). The majority of taxa and populations showed evidence for population contractions starting between 500,000 and 100,000 years ago, assuming a mean mutation rate of 6.98.10^−10^ substitutions/year, and a generation time of 2 years [19]. The only exceptions to this general trend were *P*. *nana* and the *P*. *cooperi* population north of the GRV. The northern *P*. *cooperi* population displayed evidence for a pronounced population expansion approximately 2 mya, followed by a plateau. This scenario was supported by both analyses, however, the population expansion was not as pronounced in the Stairway analysis. In *P*. *nana*, both analyses show no strong variation in population size across time. For three species (*P*.cf. *neumanni* 1, 4 and 5), both analyses show a population increase either between 5 and 3 mya (*P*.cf. *neumanni* 1, 4) or ~10 mya (*P*.cf. *neumanni* 5), followed by a population decline in the last 500,000 to 100,000 years (Fig 4).

## Discussion

Defining discrete entities (species) along the speciation continuum is a challenging task. Recent studies have highlighted issues when defining species that are in “gray zones”, that is, where speciation is still ongoing [9, 11, 45]. To adequately study speciation, it is fundamental to first quantify the extent of genetic variation and divergence in the groups of interest [4]. This quantification benefits from using both phylogenetic and population genetics approaches that can span the various stages of speciation. Another challenge lies in appropriate sampling. Poor sampling can result in spurious inferences about genetic variation, demography, and phylogenetic relationships [4, 46]. Assessing variation at a large number of unlinked loci and at a species-wide geographical scale is pivotal for adequately carrying out these types of studies.

In this study our aim was to understand the phylogeography, demographic history, and genetic variation in the genus *Ptychadena* across the highlands of Ethiopia, in order to connect microevolutionary processes with the macroevolutionary trends that drive speciation. We accomplished this by conducting dense sampling across the entire Ethiopian Highlands, including all known biogeographic zones and habitats, in concert with sequencing thousands of loci from across the genome.

### Phylogeography and demographic history of highland *Ptychadena*

Based on our demographic and phylogenetic analyses, we propose a scenario for the diversification of *Ptychadena* in the Ethiopian highlands, in which episodes of allopatry appear to be the main driver of speciation events. The ancestor of the *cooperi* group split from the rest of the *P*. *neumanni* complex early in the diversification of the group. This split was estimated to have occurred between 5.8 and 10mya [19]. After the separation of the *cooperi* group from the *nana* + *erlangeri* groups, the lowlands of the Blue Nile River split the ancestor of *P*. *cooperi* and *P*. cf. *neumanni* 5. The later species is the only member of the *neumanni* complex north of the Blue Nile, and we found no genetic structure between samples from across the range of the species, which is found as far north as the Simien Mountains (S1 Fig). The Blue Nile River has played an important role as a biogeographic barrier in the diversification of other taxa; the tree frog *Leptopelis yaldeni* is endemic to the highlands north of the Blue Nile, and this river has shaped the genetic structure of two other frogs (*Amietia nutti* and *Xenopus clivii* [47, 48]), as well as that of several mammals, including the Ethiopian wolf, the gelada baboon and rodents of the genera *Otomys* and *Stenocephalemys* [49–52]. Colonization of new areas seems to be the main driver of genetic differentiation in *P*. *cooperi*, as the population west of the GRV probably evolved from an ancestor that originated in the eastern highlands. This scenario is supported by the mtDNA analysis of Freilich *et al*. [53], who found that all western *P*. *cooperi* mitochondrial haplotypes were nested within the eastern haplotypes. Additionally, our demographic analyses show a possible bottleneck event in *P*. *cooperi* when it colonized the western highlands (Fig 4).

After their divergence from the *cooperi* group, the *nana* and *erlangeri* groups probably evolved in allopatry from one another, following the separation of their most recent common ancestor by the GRV. This hypothesis is based on the fact that most of the biological diversity of each of these two groups is found on opposite sides of the GRV (west for the *erlangeri* group and east for the *nana* group). In addition, the earliest lineages to diverge within each group are west of the GRV for the *erlangeri* group and east for the *nana* group (however, the root of the *nana* group is unresolved on the ML phylogeny). Members of both lineages subsequently crossed the GRV at later dates, after the original divergence of the two groups. This scenario is in contrast to that proposed by Freilich *et al*. [19], who suggested that the *nana* and *erlangeri* groups diverged from each other through niche diversification at the time of the Miocene-Pliocene junction. This assumption was partially based in the non-overlap in the elevational distribution between the groups and an incomplete population sampling for both the *nana* and *erlangeri* group.

Following the original split between the *nana* and *erlangeri* groups, additional migration followed by isolation must have occurred to account for the geographic distribution of the lineages constitutive of each group. However, the reconstruction of this scenario is dependent on the accuracy of our phylogenetic inference and in particular on the position of the root for each group. In the *nana* group, the ML and the SVDquartets analyses give conflicting results. In the ML topology (Figs 1 and 2) the earliest split separates *P*. cf. *neumanni* 2 and *P*. cf. *neumanni* Gecha from the three eastern lineages (although with no statistical support), while the SVDquartets analysis firmly root the group in the east, placing *P*. *nana* as the earliest lineage to diverge, while the western populations are nested within the eastern lineages (Fig 3). Whichever the case, the number of migration events across the GRV remain the same (two) for both scenarios. If the ML tree is correct, an early migration from east to west by the ancestor of the *P*.cf. *neumanni* 2 + *P*. cf. *neumanni* Gecha was followed by a more recent migration of *P*.cf. *neumanni* 2 to the east. If the SVDquartets tree is correct, the migration event of the ancestor of *P*.cf. *neumanni* 2 + *P*. cf. *neumanni* Gecha to the east occurred later in the history of the *nana* group, either with two independent migration events from east to west (leading first to the *P*. cf. *neumanni* Gecha lineage and then to the western populations of *P*.cf. *neumanni* 2) or a migration from the east to the west, and a second migration from west to east by the ancestor of *P*. cf. *neumanni* 2 in the eastern highlands.

In the *erlangeri* group, the minimum number of dispersal events across the GRV is three. In this group, three lineages are represented in the east: *P*. *harenna*, *P*. cf. *neumanni* 4 and populations of *P*. *erlangeri*. The populations of *P*. *erlangeri* from either side of the GRV are not genetically distinct from each other, suggesting current gene flow across the GRV, and the eastern samples are mitochondrially nested within western haplotypes, indicating a recent west to east migration [53]. In addition to the recent spread of *P*. *erlangeri* across the GRV, the ML and the SVDquartets phylogenies imply two migrations from west to east, one leading to *P*. *harenna* and one leading to *P*. cf. *neumanni* 4. Altogether our phylogenetic reconstructions indicate that species of *Ptychadena* have crossed the GRV on multiple occasions during the Pliocene and Pleistocene, and that each event of migration was followed by isolation, genetic differentiation and, in some cases, speciation. Thus it appears that the GRV acted as the main driver of speciation in this group. We speculate that migration across the GRV occurred during climatic periods that were colder and wetter, and when the climate became drier and hotter (as it is now), isolation and speciation took place. This scenario is consistent with what is known of the climate of east Africa over the last 8my. Palynological and paleontological analyses demonstrated that the climate of east Africa has been remarkably unstable, showing that cold and wet periods alternated with dry and hot periods [54]. These climatic changes, combined with the presence of a major topographic barrier (the GRV), constitute the perfect circumstances for the generation of biodiversity at different levels, which probably correspond to different episodes of climatic oscillations. Our scenario is consistent with a number of studies which demonstrated an important role of the GRV as a biogeographic barrier, in particular in frogs [47, 48, 53, 55], mammals [49–52], and plants [56].

The GRV does not explain the divergence of lineages that occupy the same highlands on either side of the GRV. In the *nana* group, three lineages are restricted to the eastern highlands: *P*. *nana*, *P*. cf. *neumanni* 3 and *P*. *neumanni* Mt Gugu. These lineages tend to be restricted to grassland areas at elevations from 2,400 to 3400m. It is possible that the ancestor of these three lineages was more widespread throughout the eastern highlands, but the reduction of Afro-alpine habitats since the Pliocene probably separated these populations, which were thus limited to the highest elevations, i.e. Mt. Gugu and the Bale Mountains. A similar pattern has been reported for other taxa. For example, the giant Lobelias (*Lobelia giberroa*) and the Ethiopian wolf (*Canis simensis*) occur in the same disjunct areas as these three frog species, and their distribution and genetic diversity have been affected by periods of warmer climate and habitat fragmentation [49, 56]. The Bale Mountains have experienced multiple episodes of glaciation [57], which lowered the current vegetation limit by about 1,000 m [17]. We hypothesize that the range of the ancestor of these two species was split during these periods of glaciation, and that they reconnected after the recession of glaciers, which started approximately 13,000 to 14,000 years before present [58]. The ranges of *P*. *nana* and *P*. cf. *neumanni* 3 overlap near the town of Dinsho, but the two species appear to be mostly confined to the eastern and western sides of the Bale Mountains, respectively (S2 Fig). Hybridization occurs between these two lineages, but appears to be rare, which would suggest some form of reproductive isolation (Fig 2; [19]).

In the *erlangeri* group, four lineages differentiated west of the GRV (*P*. *erlangeri sensu stricto*, *P*. cf. *erlangeri* Gecha, *P*. *erlangeri* Metu and *P*. cf. *neumanni* 1). All these lineages occur at elevations below 2,500m (S3 Fig; S1 Table) and seem to favor forested habitats (with the exception of *P*. cf. *neumanni 1* which is also found in grassland habitats under 2,500m). We propose that the ancestor of these lineages was a forest species that was widespread across the southwest of Ethiopia, when the climate of east Africa was colder and more humid. The wet-dry climatic cycles of the Pliocene and Pleistocene could have fragmented the original forest, resulting in isolation, genetic differentiation and speciation. When forest habitats were more widespread, species dispersed, resulting in overlapping distributions as observed today. It is noteworthy that these episodes of dispersal were not followed by extensive gene flow, suggesting the presence of reproductive barriers among those taxa. This scenario emphasizes the role of the forests of the Ethiopian southwest as an important source of biodiversity, which deserve urgent conservation. Multiple lineages of endemic amphibians occur in the same areas as the members of the *erlangeri* group; for example, the caecilian *Sylvacaecilia grandisonae* is endemic to the same forests in SW Ethiopia while the population of the tree frog *Leptopelis gramineus* from this region is genetically distinct from the rest of Ethiopia, and might warrant species status [55].

In addition of inferring genetically distinct units, our genome-wide SNP dataset allows us to make demographic inferences in these groups of frogs, which in turn can shed light about the role of multiple climatic and geologic events in shaping the diversification of the group. The demographic analyses of the *P*. *neumanni* species complex suggest that a continuous reduction of population size occurred in most species of highland *Ptychadena*. In the majority of taxa, our results show a constant decline in population size, which started between 500,000 and 100,000 years. These results are consistent with a constant decline of suitable habitats or high stochasticity in environmental conditions over the last half million years, which would impair population recovery. However, our demographic estimates are in contrast to those of Freilich et *al*. [53], who showed an increase in population size in several species of *Ptychadena* since the Pleistocene. Multiple cycles of glaciation in northern latitudes during the last 3 million years resulted in periods of aridity in eastern Africa, which have been linked to the evolution of African hominids [54]. These glacial cycles have played a major role in shaping the distribution and demography of a multitude of taxa, including plants [56], birds [59], mammals [49] and frogs [55]. We hypothesize that these glacial-interglacial cycles might be involved in the continuous reduction in population size across most species in our study, as well as in isolating many of the different populations.

### Evolutionary patterns, processes, and speciation in *Ptychadena*

Our study shows that multiple species and populations of *Ptychadena* from the Ethiopian highlands are at different stages of the speciation continuum. We find cases where little to no genetic variation can be found across proposed geographic barriers, such as in *P*. *erlangeri* populations across the GRV. We find other instances in which particular species show population structure across a geographic barrier, as in both *P*. *cooperi* and *P*. cf. *neumanni 2* across the GRV, but with no obvious morphological differences. In other cases, speciation appears to be at later stages, with little to no hybridization between taxa occurring in sympatry, which is one of the most universally recognized properties of biological species [60]. The only case that we have found where members of the same species groups co-occur and interbreed is in the Bale Mountains National Park, near the town of Dinsho. In this area *P*. cf. *neumanni* 3 and *P*. *nana* occur in sympatry, and we found one individual (15.163) that appears to be a hybrid between the two species. A similar case was found by Freilich *et al*. [19]. However, the lack of a wide hybrid zone between these two taxa might be the result of effective reproductive isolation. In other cases, reproductive isolation appears complete, with a total lack of hybridization. For example, the species *P*. *cooperi* is found in the highlands east and west of the GRV, where it is found in sympatry with several other members of *Ptychadena*. In the western highlands we found *P*. cf. *neumanni* 1 and *P*. cf. *neumanni* 2 in sympatry only near the town of Holeta, west of Addis Ababa. These taxa are not each other’s closest relatives and we found no evidence of hybridization between them. It is notable that these two taxa are extremely hard to distinguish morphologically, despite their high degree of genetic divergence. In southwestern Ethiopia, near the town of Gecha, *P*. cf. *neumanni* Gecha and *P*.cf. *erlangeri* Gecha are found in sympatry, while in the *erlangeri* group there are multiple instances of species occurring in sympatry (S3 Fig), yet we only find a single case where there appears to be any hybridization between the different taxa. In this case, and individual that is nested with *P*. cf. *erlangeri* Gecha in the SNP analysis (16.344) has a *P*. *erlangeri* mtDNA haplotype (S6 Fig). In all other cases the reproductive barrier between these taxa appears complete, which might justify assigning them species status.

In their early studies, Freilich et al [19] suggested an important role of ecology as a driver of speciation in this group. Our study however suggests that allopatry, which can be considered the null hypothesis when studying the speciation process, can explain the diversification of Ethiopian highland *Ptychadena*. It remains that the altitudinal segregation of the lineages is real, although not as strict as previously proposed. In the context of the allopatry scenario proposed above, it is likely that the ancestor of the *erlangeri* group was a tropical forest species, since most taxa within this group are found in this habitat, while the ancestor of the *nana* group was a mountain grassland species. Our extensive sampling across the entire Ethiopian highlands suggest that following this early habitat specialization, which coincide with an altitudinal specialization (<2,500m for the *erlangeri* group and >2,500m for the *nana* group), some lineages have adapted to novel ecological conditions. In the *erlangeri* group, all species tend to favor forest clearings with the exception of *P*. cf. *neumanni* 1, which colonized grasslands in plateaus at elevations lower than 2,500m. In the *nana* group, all species are found in grassland habitats above 2,500m, with two exceptions. The *P*.cf. *neumanni* Gecha population is found in forest habitat at a lower elevation (~2,200) than the typical habitat of other members of the group. The other exception is *P*. cf. *neumanni* 3, which can be found at elevations as high as 3,400m in the Bale Mountains, but is also found in the Harenna forest, at elevations of about 2,395m (S2 Fig). Thus it appears that the *nana* and *erlangeri* groups have generally retained the ecological niche of their ancestor, but that adaptation to novel conditions has occurred in each lineage. Although this does not seem to be common, it emphasizes the adaptability of *Ptychadena* species to novel habitats. This genus is known for its extraordinary colonizing abilities, as is found in all habitats in sub-Saharan Africa, and has even spread to oceanic islands [15, 16].

### Issues with recent taxonomic changes in Ethiopian *Ptychadena*

Our analysis is consistent with the work of previous authors [19], who showed that the strong genetic differentiation found in highlands *Ptychadena* supports the existence of multiple undescribed species in Ethiopia. Freilich et al. [19] decided not to assign names to the multiple genetic lineages they recovered. Their decision was primarily based on the lack of morphological characters useful to diagnose the different putative species, but also because their study lacked topotypic material for several of the species (personal communication). Recently, Smith *et al*. (2017) used the same dataset provided by Freilich et al. [19] and Mengistu [61], with the inclusion of a few additional samples, and assigned names to the different lineages recovered by Freilich et al. [19]. In their article, Smith *et al*. used the multispecies coalescent as the only evidence to diagnose the multiple putative new species. This alone is a major problem for their species designations (see below), but their nomenclature also has several major flaws that we describe below.

The morphological characters that Smith *et al*. use to diagnose the new taxa almost completely overlap between species of Ethiopian *Ptychadena* (e.g. size, dorsal and ventral coloration, longitudinal ridges, etc.), and thus are not, by definition, diagnostic. The type locality of *P*. *neumanni*, restricted by Perret [62] to “Gadat, Gofa, Ethiopia”, is in the southwestern part of the country (6.33N, 36.83E), and no genetic material is available from topotypic specimens. It is then not possible to assign the name *P*. *neumanni* to individuals that group with *P*. cf. *neumanni* 1, as suggested by the authors, as this taxon is not known from the type locality of *P*. *neumanni* (Fig 2, right panel; S2 Fig and S1 Table). The issue is further complicated because the type series of *P*. *neumanni* included several specimens that were later described as *P*. *nana* by Perret [62], and it is possible that individuals of other species are also represented in the type series (personal observation). Additionally, Smith *et al*. resurrected the name *P*. *largeni* Perret, 1994 as a distinct species, and assigned all specimens that grouped with *P*. cf. *neumanni 2* to this taxon, without specifying their reason to do so. The area around Addis Ababa, which is the type locality of *P*. *largeni* [63], harbors two different taxa (*P*. cf. *neumanni 1* and *P*. cf. *neumanni 2*; Fig 2, right panel, S1 Fig and S1 Table). Unless a morphological comparison is carried out, or DNA from the holotype of *P*. *largeni* is extracted and analyzed, it is impossible to confidently assign the name *P*. *largeni* to either *P*. cf. *neumanni* 1 or *P*. cf. *neumanni* 2. The taxonomy of the group is further complicated because *P*. *nana* does not have a precise type locality. Perret [62] restricted the type locality of *P*. *nana* to “Dibba”, but this can either be a town or a region in the Arsi plateau. Boehme and Roedder [64] reported an individual of this species from the town of Bekoji, while Schick [65] found individuals that he assigned to this species on the vicinity of Goba. No topotypic material is available for *P*. *nana*, so the possibility exists that in fact *P*. cf. *neumanni 3* or *P*. cf. *neumanni Mt Gugu* represent the true *P*. *nana*, and the individuals we are calling *P*. *nana* in this study, as well as those in Freilich *et al*. (2014), actually represent a different taxon.

For all of the other new species named by Smith *et al*., the authors do not provide any morphological or meristic measurements of any of the holotypes or type material. Characters that are known to aid in differentiating between the different species of *Ptychadena* were ignored (e.g. shorter legs in *P*. *nana* while longer in *P*. *erlangeri*). Smith *et al*. further describe two new lowland forms from Ethiopia (*P*. *baroensis* and *P*. *nuerensis*), but the only evidence for their decision to describe them as new is their coalescence-based species delimitation analysis, using a single mtDNA gene (16s). The use of mitochondrial loci for species delimitation has important limitations, and it has been shown that a single mitochondrial marker is not appropriate for these types of analyses and species delimitations [66].

Despite that the goal of Smith *et al*. was to help fix the taxonomic conundrum of the genus *Ptychadena* in Ethiopia, their species descriptions are inadequate, fail to abide to the rules of the Zoological Code, and only generate more confusion in this group. As a result, all new species names and combinations should, at this point, be considered *nomina nuda* according to the International Commission on Zoological Nomenclature [21] and thus disregarded until further study. Given the confusion within this group as well as the morphological similarity between taxa, a thorough taxonomic revision of Ethiopian *Ptychadena* is warranted and will certainly require the morphological (and possibly molecular) assessment of the type specimens for *P*. *neumanni*, *P*. *largeni*, *P*. *erlangeri* and *P*. *nana*.

### Important issues when applying BSD methods in Taxonomy

Since previous authors have named Ethiopian *Ptychadena* lineages with the sole justification of the multi-species coalescent, we feel it is appropriate to discuss this approach in more detail. The use of Bayesian species delimitation methods (BSD) has increased in recent years. Multiple programs for conducting BSD are now available, including BPP [67], SpedeSTEM [68], BFD* [69] and more recently PHRAPL [70], to name a few. Despite the importance of describing biological diversity of the planet, and the ability of BSD to help describe this diversity, many authors use the results of BSD analyses as the only diagnostic evidence when naming species [20, 71]. Several authors have pointed out multiple issues with the use of BSD as the only evidence for describing taxa [72, 73], but we would like to emphasize a few issues, which are particularly relevant to Ethiopian *Ptychadena*. First, it is important to note that species are by definition hypotheses, which can have different amounts of support [45, 74]; the more evidence, the stronger the support for the hypothesis. Eventually, the decision to formally describe a species lays on the taxonomist, and these decisions can be difficult to make and can have profound impacts in conservation and other areas [75]. It is also relevant to reiterate that the speciation process is a continuum, which begins with intra-population genetic variation. Sukumaran and Knowles [76] showed that methods using the multispecies coalescent are in fact delimiting genetic structure, and not species. Additionally, multiple researchers have applied BSD methods using only a few mitochondrial loci. The use of mitochondrial loci in BSD methods has important limitations, wherein the majority of cases a single mitochondrial marker is not appropriate for species delimitation [66]. This is true in amphibians in general and Ethiopian taxa in particular. For instance the populations of *P*. *cooperi* from Bale Mountains carry a highly divergent mitochondrial haplotype [53] that would surely separate them as a different species, although analysis of their nuclear genome failed to find any evidence for genetic structure with the populations on the Arsi plateau. Similarly, populations of *Leptopelis gramineus* from eastern Ethiopia show a high level of differentiation for their mitochondrial genomes, but only moderate to low differentiation when using genome-wide SNP data [55]. Lastly, the lack of appropriate sampling across a taxon’s range and not including topotypic material in BSD analyses can be problematic and is widespread among studies [46].

BSD methods can be useful in a variety of biological studies, including the naming of new taxa. This is especially true in organisms that are extremely difficult to differentiate with morphology alone, such as fungi, bacteria, and a variety of parasites [77]. However, the use of these methods should be regarded as a way to test different hypotheses, and not as the only method used to diagnose species, especially in cases where morphological differences might exist, even if they can be difficult to quantify (as in the Ethiopian *Ptychadena*). We agree with Bauer *et al*. [72] on the need to provide morphological, ecological, or other diagnostic characters when assigning new names to species, and that the assignment of a population to a particular lineage does not constitute a valid diagnostic character.

Fujita and Leaché [78] argued that finding morphological characters to define species would slow species discovery, and that multivariate analysis of morphometric data is equally as laborious as using programs such as BPP to delimit species. We strongly disagree with this view. In many instances, using BSD methods to identify species would be almost impossible for local researchers, especially in developing countries (such as Ethiopia) that lack proper resources for biological surveys. The cost associated with tissue preservation, DNA extraction, PCR amplification, sequencing, etc. is astronomical in many areas, especially when compared to local wages and university resources. If one of the main goals of naming new taxa is the conservation of those taxa [79], as stated by many supporters of BSD-only species descriptions, then these authors should provide local researchers means to properly identify these new taxa as it is the only feasible way to have an impact on the conservation of those species. It is important to point out that training in analysis of morphology or working with museum collections is becoming sort of a dying trade. Thus, the lack of diagnostic characters might be more an issue of proper training than an actual lack of diagnostic characters. We argue that the supposed conservation benefit gained by naming taxa is outweighed by the taxonomic instability and confusion that the sole use of BSD in describing taxa creates [72]. Unfortunately, we believe that the misuse of Bayesian species delimitation methods (BSD) makes it easier to name species without the need to seek further evidence to support the validity of a proposed taxonomic decision. As stated by Stephen Jay Gould [80], “Taxonomists often confuse the invention of a name with the solution of a problem”. The recent taxonomic literature on *Ptychadena* and other taxa confirms that this statement is still valid.

## Supporting information

S1 Fig Map of Ethiopia showing sample localities of *Ptychadena* in the *cooperi* group for which 16s sequence data is available. (TIF)

S2 Fig Map of Ethiopia showing sample localities of *Ptychadena* in the *nana* group for which 16s sequence data is available. (TIF)

S3 Fig Map of Ethiopia showing sample localities of *Ptychadena* in the *erlangeri* group for which 16s sequence data is available. (TIF)

S4 Fig Bayesian phylogenetic inference of members in the *cooperi* group, based on the mitochondrial gene 16s. Numbers at nodes represent posterior support. Nodes with posterior support lower than 0.5 were collapsed.
(TIF)

S5 Fig Bayesian phylogenetic inference of members in the *nana* group, based on the mitochondrial gene 16s. Numbers at nodes represent posterior support. Nodes with posterior support lower than 0.5 were collapsed.
(TIF)

S6 Fig Bayesian phylogenetic inference of members in the *erlangeri* group, based on the mitochondrial gene 16s. Numbers at nodes represent posterior support. Nodes with posterior support lower than 0.5 were collapsed.
(TIF)

S1 Table Locality information for *Ptychadena* samples used in this study. (XLSX)

S2 Table Locality information for additional samples obtained from Genbank. (XLSX)

S3 Table Output statistics from ipyrad for samples used in this study. (XLSX)

S4 Table List of adapter sequences used and order of samples in Illumina library preparation. (XLSX)

S5 Table Total numbers of fixed, shared and private polymorphisms in Ethiopian highlands *Ptychadena*, as obtained from ddRADseq analyses. (XLSX)
