# Supplementary figures and images for: Revisiting the phylogeography, demography and taxonomy of the frog genus Ptychadena in the Ethiopian highlands with the use of genome-wide SNP data
Source: PLoS One. 2018 Feb 1;13(2):e0190440. doi: 10.1371/journal.pone.0190440 (PMC5794058; doi:10.1371/journal.pone.0190440)

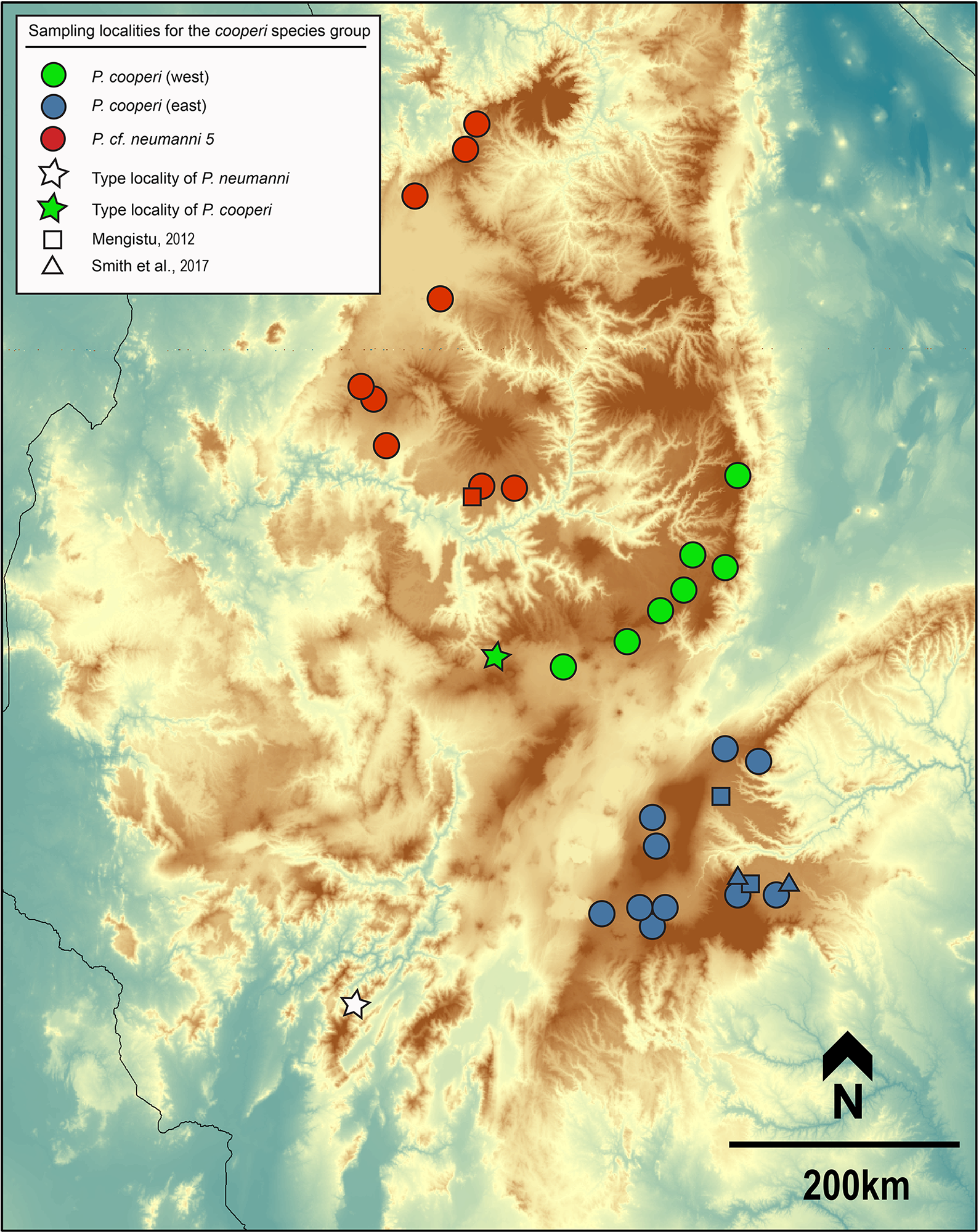

Supplement: S1 Fig — (TIF) [file pone.0190440.s001.tif]

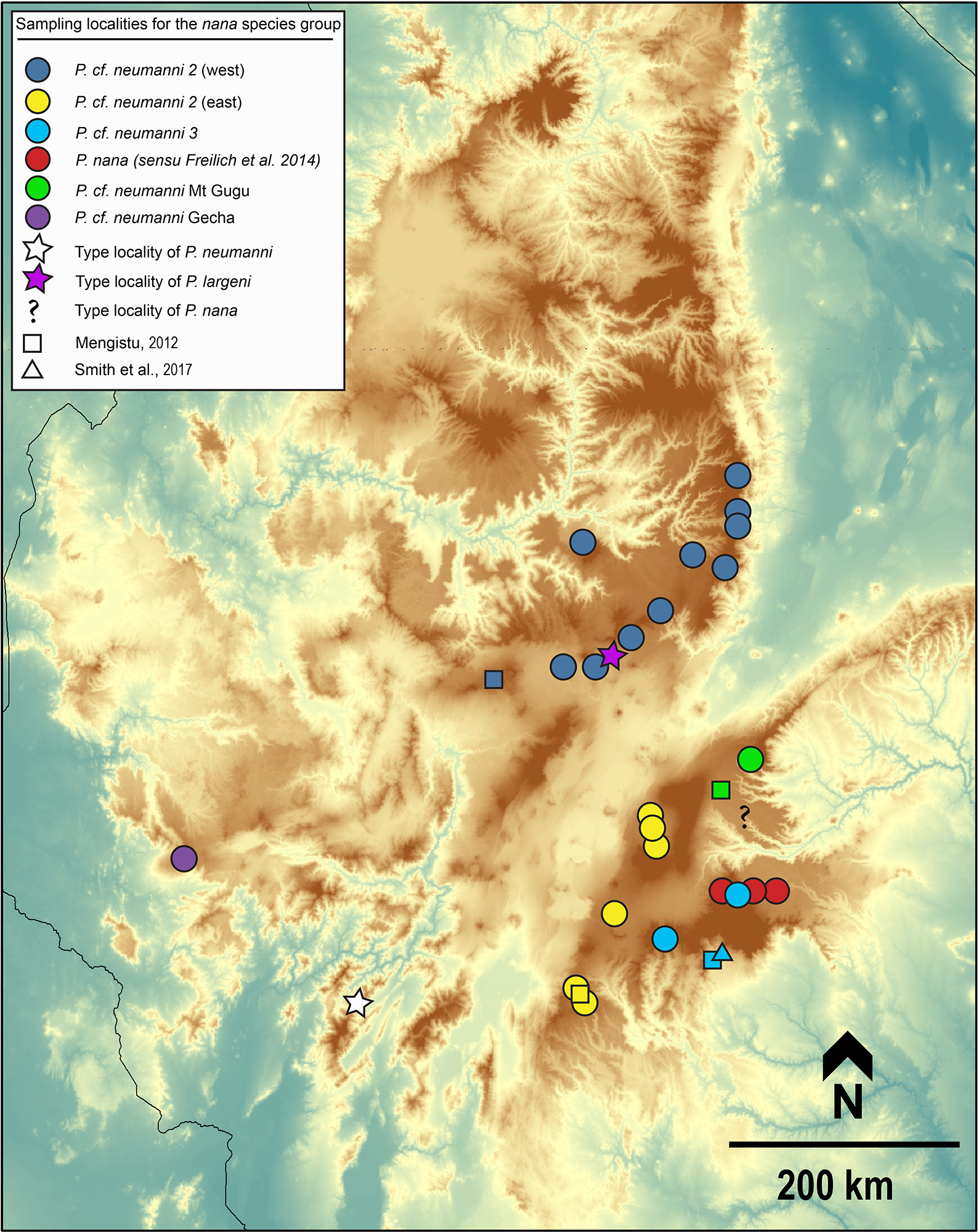

Supplement: S2 Fig — (TIF) [file pone.0190440.s002.tif]

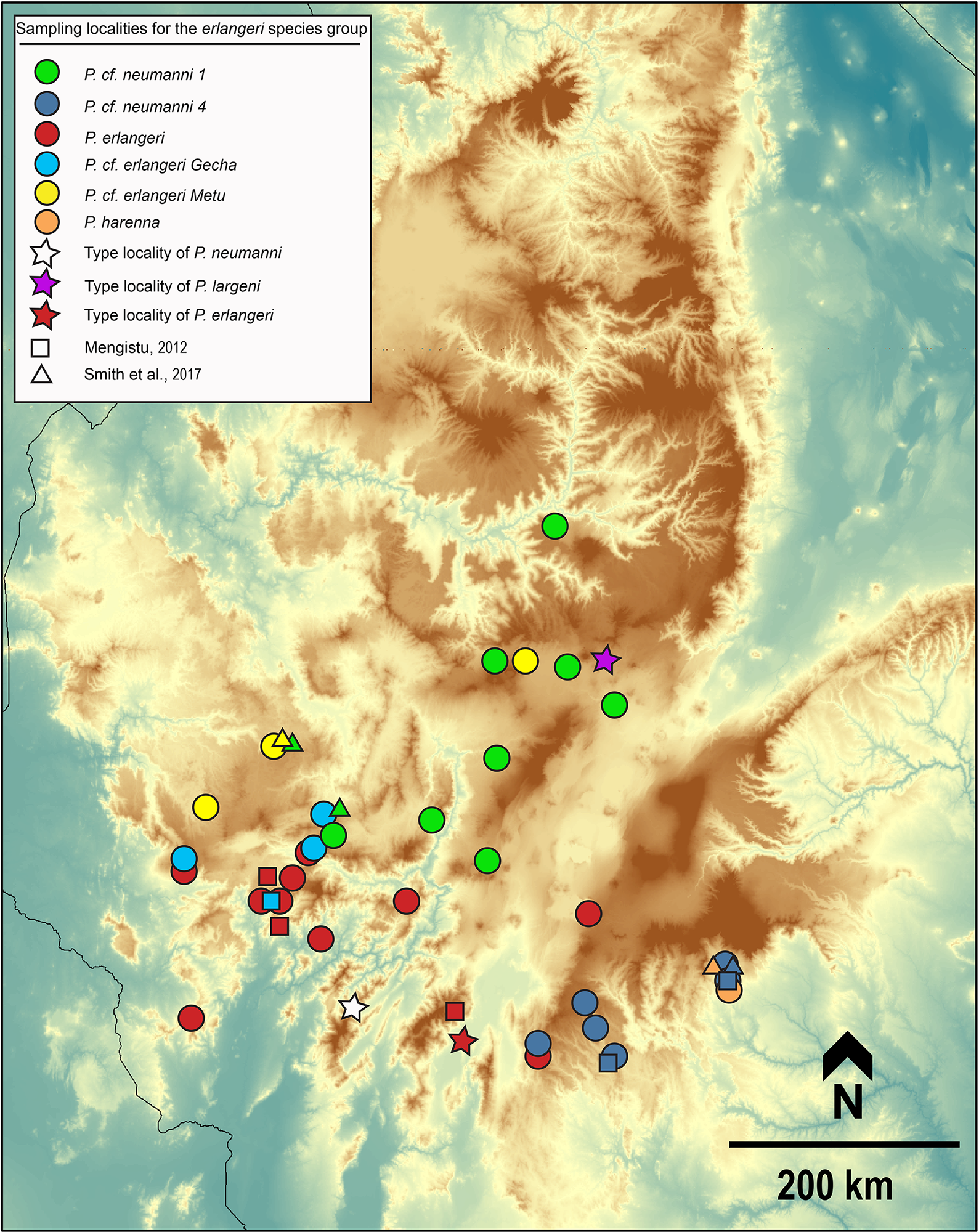

Supplement: S3 Fig — (TIF) [file pone.0190440.s003.tif]

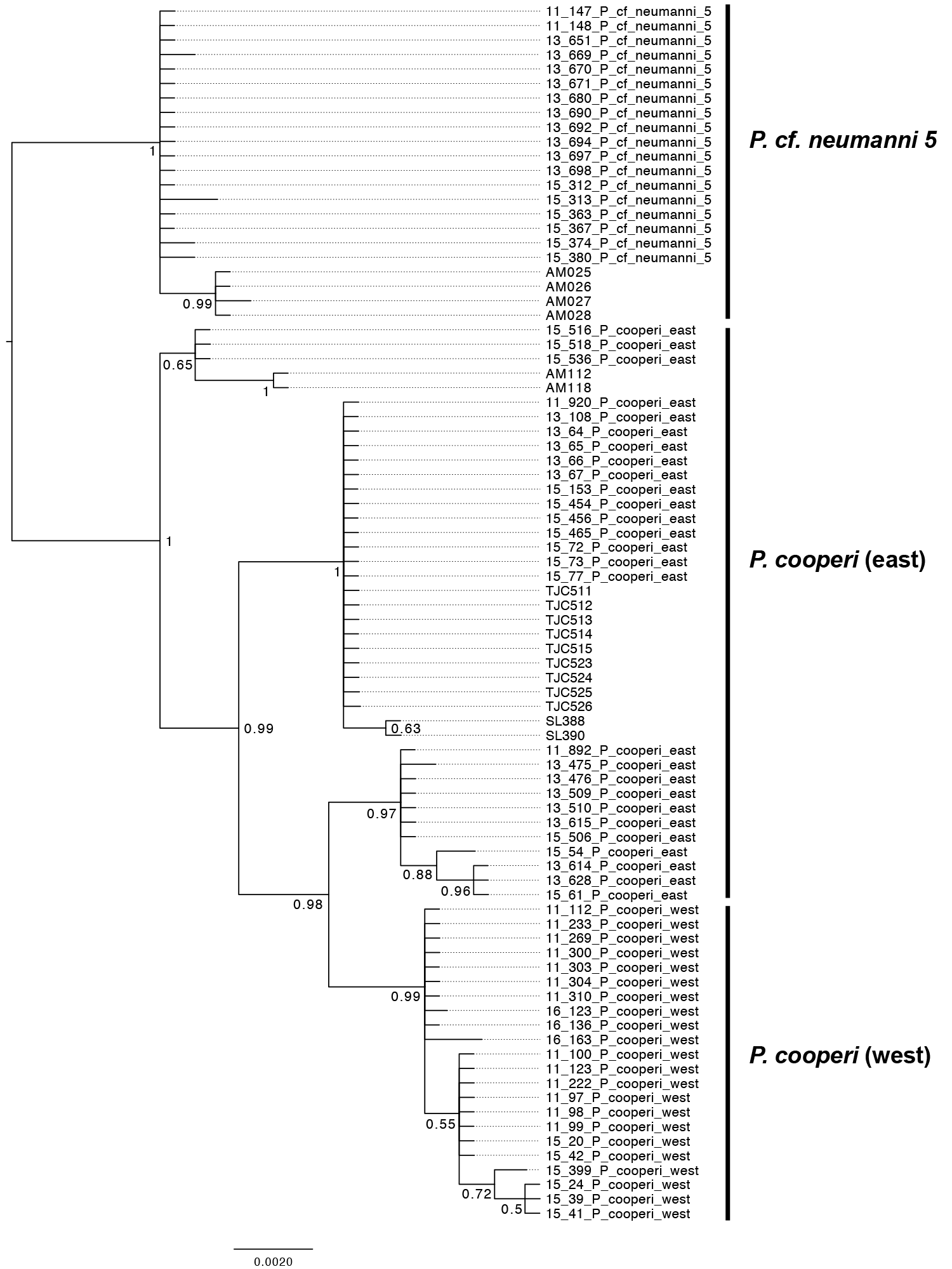

Supplement: S4 Fig — Numbers at nodes represent posterior support. Nodes with posterior support lower than 0.5 were collapsed. (TIF) [file pone.0190440.s004.tif]

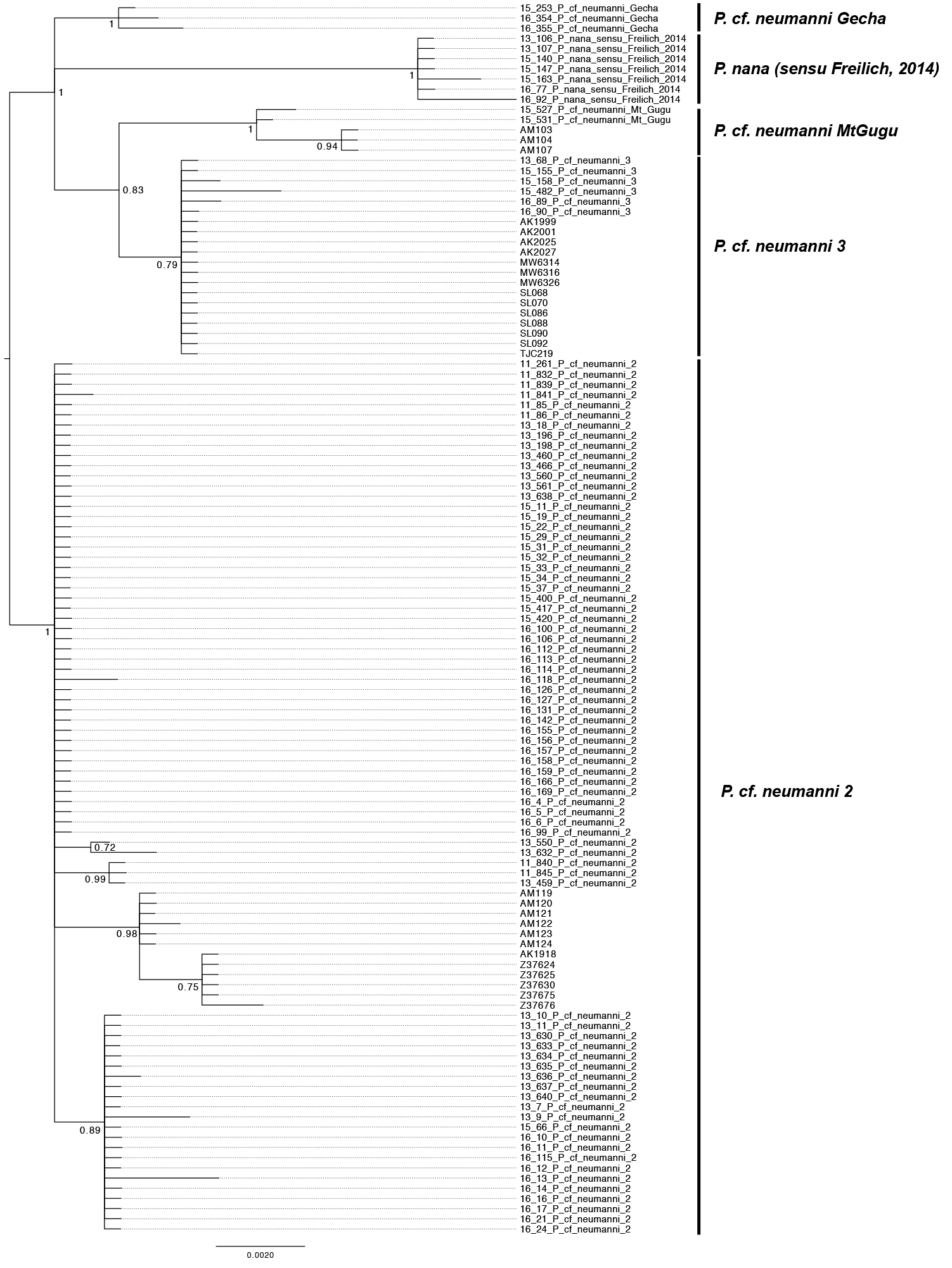

Supplement: S5 Fig — Numbers at nodes represent posterior support. Nodes with posterior support lower than 0.5 were collapsed. (TIF) [file pone.0190440.s005.tif]

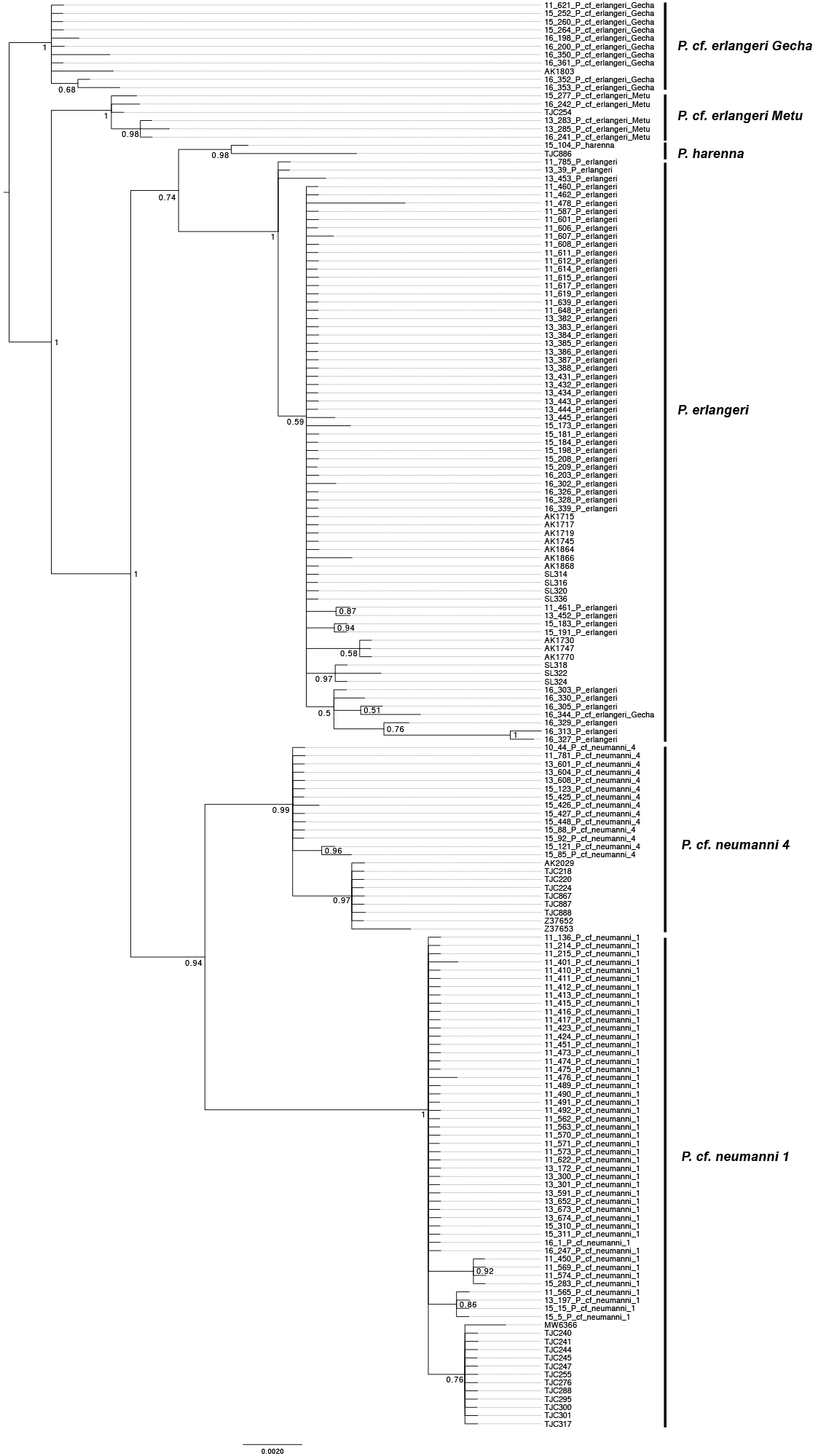

Supplement: S6 Fig — Numbers at nodes represent posterior support. Nodes with posterior support lower than 0.5 were collapsed. (TIF) [file pone.0190440.s006.tif]
